# Medication and supplement use in older people with and without intellectual disability: An observational, cross-sectional study

**DOI:** 10.1371/journal.pone.0184390

**Published:** 2017-09-06

**Authors:** Jure Peklar, Mitja Kos, Máire O’Dwyer, Mary McCarron, Philip McCallion, Rose Anne Kenny, Martin C. Henman

**Affiliations:** 1 Faculty of Pharmacy, University of Ljubljana, Ljubljana, Slovenia; 2 School of Pharmacy and Pharmaceutical Sciences, Trinity College Dublin, Dublin, Ireland; 3 IDS-TILDA School of Nursing and Midwifery, Trinity College Dublin, Dublin, Ireland; 4 Dean of the Faculty of Health Sciences, Trinity College Dublin, Dublin, Ireland; 5 School of Social Work, Temple University, Philadelphia, Pennsylvania, United States of America; 6 The Irish Longitudinal Study on Ageing (TILDA), Trinity College Dublin, Dublin, Ireland; 7 Trinity College Institute of Neuroscience (TCIN), Trinity College Dublin, Dublin, Ireland; Istituto Di Ricerche Farmacologiche Mario Negri, ITALY

## Abstract

**Introduction:**

Understanding the medication and supplement use of aging people is critical to ensuring that health service providers in primary care can optimise use of these agents. An increasing number of people with different levels of intellectual disability (ID) are living in the community and becoming for the first time substantial users of primary health care services. This, however, brings new challenges that need to be addressed at the primary health care level. We quantified the use of medicines and food supplements and described the associated patterns of morbidity in the two comparable cohorts of aging population with and without intellectual disability.

**Method:**

This research aligned participants of 50 years and over who lived in the community from two nationally representative cohorts of older people; those with ID from the Intellectual Disability Supplement (n = 238) and those without ID (n = 8,081) from the Irish Longitudinal Study on Ageing.

**Results:**

Data showed that both medication and supplement use in the two groups was prevalent but that those with ID received more of both medications and supplements (e.g. polypharmacy was 39.0% in ID vs. 18.1% in non-ID cohort). Moreover, based on an analysis of the therapeutic groups and medications used that treatment was more intense in the ID cohort (95.8 vs. 7.0 International Non-proprietary Names per 100 participants). Supplement use was almost twice as prevalent in the ID group but substantially less diverse with only 10 types of supplements reported. Morbidity was higher in the ID group and showed a higher prevalence of neurological and mental health disorders.

**Conclusion:**

The results highlight that the burden of therapy management and the potential risks in those ageing with ID differs substantially from those ageing without ID. Understanding the medication and supplement use of people aging with intellectual disability (ID) is critical to ensuring that health service providers in primary/ambulatory care can optimise use of these agents.

## Introduction

Ageing in the general population is strongly associated with multimorbidity (2 or more chronic conditions [1]), functional impairments and geriatric syndromes (falls, delirium, frailty) [2], requiring complex clinical management [3] including polypharmacy (5–9 medications) and excessive polypharmacy (≥10) [4–6]. Polypharmacy is associated with low adherence [7], adverse drug reactions [6,8]. Furthermore, with aging, people become more susceptible to adverse drug events owing to diminished physiological reserve and the accumulation of chronic conditions [9]. For the treatment of minor illness and the prevention of chronic illness and the intensification of treatment of established conditions prescription and non-prescription medications and supplements may be used on differing combinations over the lifespan. However, this adds to the complexity of the person’s medication regimens and may further increase the potential risks and occurrence of harm from adverse drug reactions and interactions in the elderly [10–11].

Intellectual disability (ID) is a disability characterised by significant limitations in both intellectual functioning and in adaptive behaviours, which covers many everyday social and practical skills [12]. Delayed development and long term marked deficits in intellectual functioning characterised by impaired communication, social skills and motor impairments are among the problems posed by the disorder [13]. Intellectual disability begins during the developmental period and it often co-occurs with other mental conditions such as depression, autism spectrum disorder, and attention-deficit-hyperactivity disorder [12]. Indeed, due to comorbidities and challenging behaviours people with ID have on average up to 2.5 times the number of health problems reported for the general population [14–16] and they are one of the most heavily medicated groups in society [16].

In line with general population trends, the life expectancy of people with ID has increased significantly, however, it remains lower than that of the general population [17–18]. Older adults with ID may be particularly challenged as a result of premature aging, age-related decline in health, increased dependency and a further decline in cognitive function, which may reduce an individual’s ability to cope, adapt and maintain quality of life [19]. Recent research in the same Irish cohort of people with ID as presented in this paper has shown, that 90% of participants reported medication use; 31.5% polypharmacy and 20.1% excessive polypharmacy [20].

From the mid-19^th^ century, care for people with intellectual disabilities in Ireland was mainly in hands of religious orders who maintained special residential centres. From the 1950s, the development of community‐based services began to emerge and from 1980 a stronger policy towards community inclusion started [21]. An increasing number of people with different levels of ID are living in the community and becoming for the first time substantial users of Primary Care services (e.g. GP, community pharmacy). This, however, brings new challenges that need to be addressed in Primary Care so that the special needs of this population may be met.

The aim of this study was to describe the patterns of use of medications and supplements and the associated patterns of morbidity in two groups of older, community dwelling people, those with, and without, Intellectual Disability since these groups have not been compared before in Ireland.

## Methods

### Study design and population

The data for this study was drawn from two sources: the Irish Longitudinal Study on Ageing (TILDA) study and the Intellectual Disability Supplement to TILDA (IDS TILDA). To facilitate comparison the groups were aligned on the basis of age—those over 50 years from each cohort; and living arrangements—those living independently or in Community Group Homes from the IDS TILDA cohort and the participants of the TILDA cohort, all of whom were living in the community. Thus, two similar groups were formed that were aligned at the group level.

The same measure for depression (moderate and severe) was used in both groups–the Centre for Epidemiologic Studies Depression Scale [22].

### TILDA

The Irish Longitudinal Study on Ageing (TILDA) is a validated national prospective cohort study that describes the social (social network, home-care), economic (income, employment, life standard) and health status (physical and mental health, medication and food supplement use, need of health services and its utilisation) of older Irish adults. More details about the study cohort profile have been published elsewhere [23].

Participants were selected by means of a three stage process of RANSAM sampling procedure [23]. Inclusion criteria for participation in this study were age 50 years or older and being resident in the Republic of Ireland. Institutionalized people were excluded from recruitment.

The data used here were taken from the first wave of TILDA from October 2009 to February 2011. Medication and supplement use data were collected during the in-home interviews performed by trained interviewers using Computer-Aided Personal Interview software (Quancept SPSS^®^). The in-home inventory of medications and food supplement was conducted by asking the question “*Now I would like to record all medications that you take on a regular basis*, *like every day or every week*. *This will include prescription and non-prescription medications*, *over-the-counter medications*, *vitamins*, *and herbal and alternative medications*.” No information about dose, frequency and quantity or prescription status was obtained.

A total of 8,175 people participated in but in 94 cases data on medication and supplement use were not collected (participants didn’t want to give or didn’t know the details about the medications/ supplements use during the interview) which corresponds to a participation rate of 98.9%. The TILDA study was approved by the research ethics committee of Trinity College Dublin, and subjects were required to sign an informed consent document prior to participation.

### IDS TILDA

Data on older adults with intellectual disabilities was drawn from the first wave of data collected between 2009 and 2010 for the Intellectual Disability Supplement to the Irish Longitudinal Study on Ageing (IDS TILDA). The sample was randomly selected from Ireland’s National Intellectual Disability Database (NIDD) which collects information on 26,000 people with all levels of ID, eligible for or receiving services and in a full range of residential circumstances [24]. This study has been described in detail elsewhere [25–26]. Inclusion criteria were: age ≥40 years with intellectual disability and written consent to participate and/or family/guardian written agreement, where required. The recruited, consented and protocols completed sample was 753 persons with an ID, aged between 41 and 90 years and represented 8.9% of the total population aged 40 and over registered on the 2009 NIDD database. A pre-interview questionnaire (PIQ) was sent to each participant/ carer before a formal in-person interview. The PIQ covers demographical information including age, level of ID (Down syndrome or ID from other aetiology) physical and mental health status (including physician confirmed diagnoses of physical and mental health concerns), healthcare utilisation and medication usage taken on a regular basis (‘every day or every week’, as in TILDA). Additional data was gathered in a subsequent face to face interview when PIQ reports including the information on the regular use of medications and supplements was cross-checked and verified for 736 (97.7%) participants. For the purpose of this paper the cohort of participants living in the community aged 50 years and over was only used to be aligned with the TILDA cohort inclusion criteria. This group comprised 238 participants for whom data on medications and health status was available. Medication usage included drugs recorded by brand/generic name, including prescription and non-prescription herbal and alternative drugs and any supplements.

The IDS TILDA study was approved by the research ethics committee of Trinity College Dublin and all of the participating services providers (n = 138).

The aim of this study was to describe the patterns of medication and supplement use in two groups of older, community dwelling people and to facilitate this comparison the groups were aligned on the basis of age, those over 50 years from each cohort, and living arrangements, those living independently or in Community Group Homes (IDS TILDA) whereas those living in residential care facilities are excluded from the TILDA cohort and were not included from the IDS TILDA cohort records. Residents of residential facilities and nursing homes are known to receive more medications than those living in the community both in the elderly [27] and in older people with ID [20].

### Medication and supplement data collection

A supplement was defined according to the Directive 2002/46/EC of the European Parliament and of the Council, of 10 June 2002: “*Food supplements* means foodstuffs the purpose of which is to supplement the normal diet and … other substances with a nutritional or physiological effect. . . .” [28].

Medication data was coded using the ATC (Anatomical Therapeutic Chemical) classification system [29] which uses five levels from body system to drug name (International Non-Proprietary Name) to categorise each type of preparation.

Products with ATC groups C10AX06 (omega-3-triglycerides), M01AX05 (glucosamine), A12AA (calcium), A11D, A11E and B03B (vitamins B), B03A (iron), A11A and A11B (multivitamins with minerals), A11GA01 (vitamin C) and A11CC (vitamin D and analogues) were classed as supplements.

Morbidity, medication and supplement use was compared across genders and age groups (50–59, 60–69 and ≥70 years) both the TILDA and IDS TILDA. Due to large difference in number of participants in the cohorts, number of medications and supplements were compared using same denominator (“per 100 enrolled participants”).

### Statistical analysis

Descriptive statistics were used to illustrate differences in the prevalence of medication and supplement use, morbidity and clinical characteristics of both populations. Prevalences were presented as percentages including 95% confidence intervals (CI). Group differences for each cohort were assessed by Pearson’s chi-square test for categorical variables and analysis of variance (ANOVA) for continuous ones. Two-sided significance tests were used throughout and p-value <0.05 were considered statistically significant.

To control for problems associated with multiple comparisons (which increases the likelihood of rejecting the null hypothesis when it is true; Type I error), and the False Discovery Rate (FRD) we adopted the Bonferroni correction procedure to maintain the Family wise Error Rate (FWER) [30]. In relation to demographic and clinical characteristics, we tested 20 and 21 hypotheses in the TILDA and IDS TILDA, respectively, with a desired α of 0.05, the Bonferroni correction tested each individual hypothesis at α = 0.05/20 = 0.0025 and α = 0.05/21 = 0.0024, respectively.

All analyses were performed using SPSS 20 (SPSS for Windows Release 20.0).

## Results

### TILDA and IDS TILDA cohorts

The age and sex profiles of the two cohorts presented in Table 1 differ and are consistent with the shorter life expectancy of people with ID and the preponderance of men in this population. Both medication and supplement use was substantially higher in the IDS group (73.1 IDS TILDA vs 49.5% TILDA respectively) in line with greater multimorbidity. The range of reported conditions was 0 to 9 in the TILDA group and 0 to 7 in IDS TILDA. However, the pattern of chronic conditions varied with a substantially greater prevalence in the TILDA group of hypertension (36.9% vs. 21.0%), bone and joint disease (33.3% vs. 21.4%), cancer (6.3% vs 4.6%) and moderate to severe depression (27.2% vs. 14.7%) while conversely, gastro-intestinal conditions (peptic ulcer, constipation; 17.2% vs. 7.8%) and eye conditions were more prevalent in the IDS group (67.6% IDS TILDA vs. 16.4% TILDA). Information on other mental health conditions (39.5%) and on epilepsy (24.1%) was collected only in IDS TILDA.

**Table 1:** Comparison of the demographic and clinical characteristics (not all characteristics have been explored equally in both groups).

TILDA		IDS TILDA	
N (%) 8,081	**Respondents**		N (%) 238
3,706 (45.9%)	**Male**		134 (56.3%)
4,375 (54.1%)	**Female**		104 (43.7%)
	**Age**		
3,256 (40.3)	50–59		152 (63.9)
2,563 (31.7)	60–69		67 (28.2)
2,262 (28.0)	≥70		18 (8.0)
5,622 (69.6)	Education (≥ secondary school)		
	**Disability**^**#**^	**Level of ID**^**$**^ **[missing 26]**	
275 (3.4)	IADL disability	Mild	76 (35.8)
687 (8.5)	Any ADL disability	Moderate	109 (51.4)
		Severe/Profound	27 (12.8)
	**Living status**		
1,782 (22.1)	Living alone	Independent/Family	69 (29.0)
		Community/Group Home	169 (71.0)
2.35 (±2.55)	**Number of medications (mean+SD)**		4.55 (±3.87)
1,405 (17.4)	**Any supplement reported (%)**		80 (33.6)
0.25 (0.64)	**Number of supplements (mean+SD)**		0.47 (±0.76)
1,239 (15.4) [missing = 13]	**Self-rated health [Fair/Poor] (%)**		31 (13.1) [missing = 2]
4,002 (49.5)	**Multimorbidity** **(≥2 chronic conditions)**		174 (73.1)
1.71 (1.45))	**Number of chronic conditions [mean+SD]**		2.51 (±1.43)
2,981 (36.9)	**Hypertension**		50 (21.0)
1,165 (14.4)	**Heart disease** **(angina, heart attack, AF, heart failure)**		27 (11.3)
286 (3.5)	**Stroke (Stroke and TIA)**		6 (2.5)
621 (7.7)	**Diabetes**		29 (12.4) [missing = 4]
1,326 (16.4)	**Eye disorders **		161 (67.6)
2,691 (33.3)	**Bone/joint disease (osteoporosis, arthritis)**		51 (21.4)
512 (6.3)	**Cancer**		11 (4.6)
325 (4.0)	**Lung disease (COPD, asthma)**		16 (6.7)
634 (7.8)	**Peptic ulcer, constipation**		41 (17.2)
2,160 (27.2) [missing = 126]	**Depression (severe/moderate)**		35 (14.7)
		**Mental health & neurological** **disorders** ^*^	94 (39.5)
		(schizophrenia, psychosis, depression)	
		Epilepsy [missing = 1]	57 (24.1)

^#^ADL and IADL (basic and instrumental activities of daily living) are measures of one’s independent living skills and is measured with the questionnaire

^$^ Level of Intellectual Disability indicates both intellectual and adaptive functioning deficits in conceptual, social, and practical domains.

* Data on these conditions were not available in TILDA cohort

### Use of medications and supplements

Table 2 illustrates that use of medications and supplements in the IDS TILDA group was greater than in the TILDA group; however, the most notable difference was in the degree of excessive polypharmacy which was 6 times more prevalent (TILDA 1.9% vs 11.3% IDS TILDA). Furthermore, use of a supplement with at least one medication, was over twice as prevalent in the IDS group (14.6% TILDA vs 32.8% IDS TILDA). The mean number of medications in both groups grew as age increased but the increase was greater in the TILDA group and the extent of the difference between the two groups diminished with increasing age. Supplement use also increased with age in both groups but in the IDS TILDA group the increase with age was greater and the disparity between the two cohorts also grew. Women used more medications and more supplements than men within both TILDA and IDS TILDA and the differences were more marked within IDS TILDA than in TILDA.

**Table 2:** Medication and supplement use compared.

	TILDA (n = 8,081)	IDS TILDA (n = 238)
At least 1 medication	5,572 (69.2%)	212 (89.1%)
5–9 medications	1,309 (16.2%)	66 (27.7%)
≥ 10 medications	154 (1.9%)	27 (11.3%)
At least 1 supplement	1,404 (17.4%)	80 (33.6%)
Concomitant use	1,180 (14.6%)	78 (32.8%)
Medication use per chronic condition (Mean No of medications±SD)	1.34 (±1.24)	2.06 (±1.68)
Medication use with age (Mean No of medications±SD)	2.35 (±2.55)	4.55 (±3.87)
50–59	1.43 (±2.04)	3.95 (±3.68)
60–69	2.39 (±2.47)	5.46 (±4.11)
70+	3.62 (±2.73)	6.16 (±3.53)
Supplement use with age (Mean No of supplements ±SD)		
50–59	0.2 (±0.64)	0.43 (±0.77)
60–69	0.26 (±0.65)	0.51 (±0.77)
70+	0.29 (±0.64)	0.68 (±0.67)
Medication use by sex (Mean No of medications ±SD)		
Male	2.31 (±2.60)	4.15 (±3.75)
Female	2.38 (±2.49)	4.87 (±3.94)
Supplement use by sex (Mean No of supplements±SD)		
Male	0.14 (±0.48)	0.25 (±0.52)
Female	0.34 (±0.74)	0.65 (±0.87)
No of chronic conditions	Combined medication and supplement use (Mean No of combined medications and supplements ±SD)	
0	0.66 (±1.27)	1.00 (±1.15)
1	1.67 (±1.81)	2.96 (±2.78)
2	2.86 (±2.17)	3.58 (±3.27)
3	4.16 (±2.58)	6.57 (±4.08)
4	5.51 (±2.79)	6.26 (±3.86)
5	6.50 (±3.00)	9.14 (±3.42)
6	7.85 (±2.93)	11.8 (±5.40)
7	7.69 (±4.20)	10.5 (±7.78)
8	9.78 (±4.27)	-

### Types of medications and supplements used

The diversity of reported medications (Table 3 and Table 4) and supplements (Table 5) differs between the cohorts with 568 different drugs and 134 different supplements in TILDA and only 228 drugs and merely 10 supplements in IDS TILDA. However, when the number of participants is taken into account (568/8,081 TILDA vs 228/238 IDS TILDA) there was much higher diversity of drug use in the IDS cohort: more than 13-times (TILDA 7.0 vs. IDS TILDA 95.8 drugs per 100 participants).

**Table 3:** Reported number of medications and their INNs (ATC 5^th^ level).

TILDA [n = 8,081]				Therapeutic class	IDS TILDA [n = 238]			
Medications/100 enrolled participants	N of medications	Different drugs/100 enrolled participants	Different drugs	1^st^ ATC level	Different drugs	Different drugs/100 enrolled participants	N of medications	Medications/100 enrolled participants
34.7	2,807	1.0	78	**A—Alimentary**	40	16.8	256	107.6
27.4	2,211	0.2	13	**B- Blood**	3	1.3	28	11.8
100.4	8,110	1.3	107	**C- Cardiovascular**	36	15.1	167	70.2
1.2	95	0.4	32	**D- Dermatology**	15	6.3	40	16.8
6.7	545	0.5	41	**G- Genito-Urinary**	11	4.6	19	8.0
9.2	744	0.2	15	**H- Hormones**	7	2.9	41	17.2
1.8	143	0.4	33	**J–Anti-infective**	7	2.9	12	5.0
3.0	244	0.3	27	**L–Malignancy & Immuno- modulation**	1	0.4	2	0.8
16.1	1,299	0.5	39	**M- Musculo-Skeletal**	11	4.6	43	18.1
32.4	2,617	1.3	105	**N- Nervous system**	61	25.6	486	204.2
0.1	6	0.0	3	**P—Antiparasitic**	1	0.4	1	0.4
15.0	1,214	0.6	45	**R- Respiratory**	21	8.8	72	30.3
2.9	238	0.3	27	**S- Sensory**	11	4.6	21	8.8
0.1	8	0.0	3	**V- Various**	3	1.3	10	4.2
**251.0**	20,281	**7.0**	568	**All**	228	**95.8**	1,198	**503.4**

**Table 4:** Comparison of the 20 most frequently reported therapeutic classes (2^nd^ ATC level).

	IDS TILDA		TILDA	
Most frequently reported medications by ATC (2nd level)	Rank	%	%	Rank
N05 Psycholeptics (antipsychotics, anxiolytics, hypnotics)	1	11.0	3.1	11
N06 Psychoanaleptics (antidepressants, stimulants)	2	6.1	3.0	12
R03 Drugs for obstructive airway diseases	2	6.1	4.8	6
C09 Agents acting on renin-angiotensin system	4	5.3	10.2	3
N03 Antiepileptics	5	4.8	1.3	18
A06 Drugs for constipation	6	4.4	0.5	25
S01 Ophtalmologicals	6	4.4	1.1	20
A02 Drugs for acid related disorders	8	3.9	6.2	4
A10 Drugs used in diabetes	9	3.1	3.6	9
G04 Urologicals	9	3.1	1.7	16
N02 Analgesics	9	3.1	3.0	12
C10 Lipid modifying agents	12	2.6	13.9	1
D01 Antifungals for dermatological use	12	2.6	0.1	37
J01 Antibacterials for systemic use	12	2.6	0.6	23
M01 Anti-inflammatories and antirheumatics	12	2.6	4.1	7
A03 Drugs for functional GI disorders	16	1.8	0.5	25
C07 Beta blocking agents	16	1.8	5.6	5
C03 Diuretics	16	1.8	3.6	9
C08 Calcium channel blockers	16	1.8	3.9	8
A07 Antidiarrhoeals	16	1.8	0.3	33

**Table 5:** Reported supplements for TILDA and IDS TILDA.

TILDA [n = 8,081]			IDS TILDA [n = 238]		
Supplements	N	Per 100 enrolled participants	Supplements	N	Per 100 enrolled participants
Calcium with or w/o D vitamin	619	7.7	Calcium with or w/o D vitamin	36	15.1
Vitamins (alone or multiple)	468	5.8	Vitamins (alone or multiple)	34	14.3
Omega-3-triglycerides	360	4.5	Iron	20	8.4
Glucosamine	263	3.3	Evening primrose oil	6	2.5
Iron	97	1.2	Omega 3	6	2.5
Evening primrose oil	56	0.7	Glucosamine	3	1.3
Garlic	23	0.3	Linseed	4	1.7
Carotenoids (lutein, meso-zeaxanthin)	23	0.3	Carotenoids (lutein, meso-zeaxanthin)	2	0.8
Echinacea	14	0.2	Acidophilus	2	0.8
Other non-ATC supplements*	171	2.1	Peppermint oil	1	0.4
**All**	**2,094**	**25.9**	**All**	**114**	**47.9**

* aloevera, starflower, cranberry, milk thistle,…

The usage was greatest in certain therapeutic groups within the IDS cohort; for conditions of the Gastro-Intestinal tract and metabolism (A)—three times as many medications and 17 times as many different drugs, Dermatological conditions (D) - 14 times as many medications and 16 times as many drugs, and Nervous system conditions (N) - 6 times as many medications and 20 times as many drugs. In contrast, the use of medications for cancer and immunomodulation–L was 73% lower overall in IDS TILDA but with more drugs in use compared to the TILDA group. Similar pattern was observed in the Cardiovascular conditions (C) group.

Comparing the 20 most frequently reported therapeutic groups (2^nd^ ATC level) in IDS-TILDA showed that 15 of them were common to TILDA cohort (Table 4). Of the 5 other therapeutic groups used in the IDS cohort, 3 were used to treat gastro-intestinal conditions whereas in the TILDA cohort, 3 of the 5 other therapeutic groups were associated with cardiovascular and haematological conditions.

In contrast to the greater variety of medications per participant identified in the IDS TILDA group, supplement utilisation illustrated minimal diversity with only 10 types of supplements in use. However, 9 supplements in the IDS TILDA group and 8 in the TILDA group were used by 1% or more of the cohort and in both, calcium with or without vitamin D and vitamins, alone or in combination preparations were the most prevalent.

The pattern of medication and supplement usage alone and in combination shows considerable variation across the range of reported conditions (Table 6). In both groups, 9 out of 10 participants reported use of either or both medications and supplements but in the IDS TILDA group only 5.7% (95% CI 3.6, 7.7) reported taking neither, whereas in the TILDA group this proportion was almost double 10.1% (95% CI 6.2, 13.9).

**Table 6:** Medication (M) and supplement (S) use and chronic conditions in TILDA (T) and IDS TILDA (IDS) cohort.

Taking/ Condition (TILDA vs. IDS TILDA)	Hypertension 36.9%: 21.0%		Heart disease (angina, heart attack, AF, heart failure) 14.4%: 11.3%		Stroke (Stroke and TIA) 3.5%: 2.5%		Diabetes mellitus 7.7%: 12.2%		Eye disorders 16.4%: 67.6%		Bone/joints disease (osteoporosis, arthritis) 33.3%: 21.4%		Cancer 6.3%: 4.6%		Lung disease 4.0%: 6.7%		Peptic ulcer 7.0%: 4.2%		Depression symptoms (severe/moderate) 26.7%: 14.7%		Mental health condition 39.5%	Epilepsy 23.9%
	T (n = 2981)	IDS (n = 50)	T (n = 1165)	IDS (n = 27)	T (n = 286)	IDS (n = 6)	T (n = 621)	IDS* (n = 29)	T (n = 1326)	IDS (n = 161)	T (n = 2691)	IDS (n = 51)	T (n = 512)	IDS (n = 11)	T (n = 325)	IDS (n = 16)	T (n = 567)	IDS (n = 10)	T ^§^ (n = 2160)	IDS (n = 35)	IDS (n = 94)	IDS^¥^ (n = 57)
**No M, no S**	201	5	59	1	9	0	23	1	149	14	334	4	68	1	38	0	103	2	462	0	5	1
	6.7%	10.0%	5.1%	3.7%	3.1%	0.0%	3.7%	3.4%	11.2%	8.7%	12.4%	7.8%	13.3%	9.1%	11.7%	0.0%	18.2%	20.0%	21.4%	0.0%	5.3%	1.8%
**M only, no S**	2219	27	853	14	199	2	498	17	833	90	1553	19	328	6	214	9	341	14	1261	27	57	29
	74.4%	54.0%	73.2%	51.9%	69.7%	33.3%	80.2%	58.6%	62.8%	55.9%	57.7%	37.3%	64.1%	54.5%	65.9%	56.3%	60.1%	63.6%	58.4%	77.1%	60.6%	50.9%
**M & S concomitantly**	542	18	241	12	77	4	95	11	323	55	734	28	105	4	70	7	112	6	388	8	32	27
	18.3%	36.0%	20.7%	44.4%	26.9%	66.7%	15.3%	37.9%	24.4%	34.2%	27.3%	54.9%	20.5%	36.4%	21.5%	43.8%	19.8%	27.3%	18.0%	22.9%	34.0%	47.4%
**S only**	19	0	12	0	1	0	5	0	21	2	70	0	11	0	3	0	11	0	49	0	0	0
	0.6%	0.0%	1.0%	0.0%	0.3%	0.0%	0.8%	0.0%	1.6%	1.2%	2.6%	0.0%	2.1%	0.0%	0.9%	0.0%	1.9%	0.0%	2.3%	0.0%	0.0%	0.0%

* Missing = 4.

§ Missing = 126.

¥ Missing = 1.

Use of supplements alone was only reported in eye disorders within the IDS group (1.2%) whereas use alone was reported in all of the conditions within the TILDA group and in particular in those reporting bone or joint disease (2.6%), depression (2.3%) or cancer (2.1%). Use of medications alone was most frequently associated with diabetes mellitus (80.2%) and hypertension (74.4%) in the TILDA group and in moderate or severe depression (77.1%) and mental health conditions (60.6%) in the IDS TILDA group.

Concomitant use was most prevalent in both cohorts in those reporting bone or joint disease (54.9%) with a calcium preparation with or without vitamin D taken with medications. By contrast the lowest concomitant usage in the TILDA group and one of the lowest in the IDS group are associated with those reporting diabetes mellitus 15.3% TILDA vs 37.9% IDS TILDA). Concomitant use was highest in the IDS group in stroke although this was the smallest subgroup (n = 6).

The use of neither medications nor supplements was frequently reported among the TILDA group most notably by those reporting moderate or severe depression (21.4%) and in 18.2% of those with peptic ulcer and 12.4% of those with bone or joint disease. Of the larger subgroups among the IDS TILDA participants, those reporting eye disorders (8.7%), hypertension (10.0%) and bone or joint disease (7.8%) also recorded neither medication nor supplement use.

## Discussion

There are few comparative studies of medication use in these populations and this is the first to describe the use of both medications and supplements. This study shows that in two similar groups of community dwelling elderly patients’ medication and supplement use was prevalent but in those with ID both medication and supplement use was higher in all age groups. The pattern of morbidity reflected, in part, the therapeutic classes used but for some, such as peptic ulcer and moderate to severe depression, 20% or more of the TILDA group reported no use of either a medication or a supplement; whereas the use of a medication and/or a supplement was almost universal in the IDS group. In the IDS group there was extensive use of medications in epilepsy, mental health conditions and depression and particularly the high level of excessive polypharmacy, may indicate more intensive drug treatment [31]. Although in the TILDA group a disparate group of medications was used, when this was standardised per 100 participants, it was clear that the IDS group was exposed to a proportionately wider range of medications. In contrast, the types of supplements used in the IDS group were extremely limited in comparison to the TILDA group suggesting that TILDA participants were prescribed and/or chose from a wider range of alternatives than those in the IDS group, although the two most frequently used classes were the same. Use of supplements alone was only significant in the TILDA group but the level of usage was low at only just over 2%.

Medications may pose some risk to most groups but some types of medication are particularly risky for the elderly. Long term use of sedative and anticholinergic medications have been associated with cognitive impairment, increased risk of falls, of hospitalisation and of hospital admission. Increased sedative load has been reported for the TILDA population with those most exposed having poorer health and sedative load was associated with being prefrail and frail [32] while in the IDS cohort (including those aged 40–49 years and those in nursing homes) a high anticholinergic burden was found and it was associated with daytime dozing and constipation [33]. This evidence strongly suggest that the groups studied in this work are exposed to risks and vulnerable to harm from both the types and the high levels of medications and supplements that they use. This reinforces the need for a systematic and structured approach to managing the level of exposure, to high risk medications and to reducing their vulnerability. Since these groups live in the community, it is most appropriate that they continue to be cared for in the community. Those Primary Care providers who share responsibility for the provision of medications and supplements, general practitioners and community pharmacists, should be supported and empowered to collaborate in the care of these diverse populations. Our findings highlight the scale and complexity of the challenge of the future management of these agents in both these populations.

For the TILDA group there is also a substantial but different composition of sources of risk from medications and supplement use. The diversity of medications and supplements used in this large group reduces the likelihood that Primary Care providers will have extensive experience in using the less frequently encountered drugs and supplements and the even rarer permutations of combined use that may occur. Previous detailed research in the TILDA population showed that 4.5% of concomitant users could suffer some type of major drug-supplement interaction [11]. Recently, considerable interest has been shown in approaches to reducing the numbers of medications used, deprescribing, in older people, particularly those receiving palliative care [34]. Nevertheless, this requires a mechanism for reviewing all of the medications and supplements and although pilot work has been carried out [35], no initiative has been brought forward by the health service in Ireland. Similarly, while providers of nursing home care are required to ensure that medication reviews are performed every 6 months, medication reviews would also benefit people with ID living in the community but the requirement for this has not been acknowledged [36].

In the IDS group the burden is the most marked because of the high use of both medications and supplements at all ages and because their capacity to take part in the management of their conditions, symptoms and treatments is limited and may diminish with ageing. Consequently, this burden is mainly carried by non-family carers because as people with ID live longer they are more likely to outlive their parents. The present and future consequences of this have not been evaluated and in Ireland, few carers receive training about medication and supplement use and unless the care providers address medications information and management issues, it is unlikely that anyone else does [37]. Furthermore, the complexity of the clinical conditions and the combination of medications and supplements used would be difficult for anyone, health professional or carer, to manage, and in many instances, such as people with epilepsy, care is provided both by specialists in Secondary Care clinics and generalists in Primary Care which adds potential communication difficulties to the care process. Thus the potential for adverse drug reactions and interactions as well as drug-supplement interactions and administration errors must be considerably greater in the IDS than in the TILDA group. Therefore as people with ID are enabled to live in the community in greater numbers suitable mechanisms for the regular review of medication and supplement use, the education of carers about the use of medications and supplements and the co-ordination of care are increasingly needed to mitigate the associated risk and to prevent harm [38].

### Strengths and limitations

Both the TILDA and IDS TILDA cohorts used robust sampling techniques to establish two nationally representative cohorts. The data collection periods were contemporaneous and almost all the participants in both cohorts recorded medical data (98.9% and 99.6% respectively), with validated response quality data capture that allowed for detailed analysis of medication and food supplement usage patterns, morbidity and socio-economical and lifestyle characteristics. Data on medication and food supplement use was collected by in-home interview (TILDA) or with a preinterview questionnaire and then validated within the face-to-face interview (IDS TILDA) which improves reliability compared to self-report recall alone [39]. The use of the Bonferroni correction addressed the problem of multiplicity. Both populations were matched regarding the age and living status.

There are also certain limitations: Only regularly taken medications and supplements were recorded and not those taken “when required” (prn). There was no data on the dose of medications or supplements or the way they were obtained (prescribed or purchased). The reporting of chronic conditions was based on participant or proxy self-report in both cohorts and thus may be prone to a misclassification bias [28] and the severity of the condition was not captured, except in the instance of depression. Matching at the level of the individual and adjustment using a technique such as propensity scoring was not undertaken because it would have required creation of a specific dataset which was not possible within the constraints of the Ethics approvals, the range of variables included, and the time available for this study.
